# Metabolomics Revealed an Association of Metabolite Changes and Defective Growth in *Methylobacterium extorquens* AM1 Overexpressing *ecm* during Growth on Methanol

**DOI:** 10.1371/journal.pone.0154043

**Published:** 2016-04-26

**Authors:** Jinyu Cui, Nathan M. Good, Bo Hu, Jing Yang, Qianwen Wang, Martin Sadilek, Song Yang

**Affiliations:** 1 School of Life Science, Qingdao Agricultural University, Shandong Province Key Laboratory of Applied Mycology, and Qingdao International Center on Microbes Utilizing Biogas, Qingdao, Shandong Province, China; 2 Department of Microbiology, University of Washington, Seattle, Washington, United States of America; 3 Kemin Industries, KI Research & Development, Des Moines, Iowa, United States of America; 4 Central Laboratory, Qingdao Agricultural University, Qingdao, Shandong Province, China; 5 Department of Chemistry, University of Washington, Seattle, Washington, United States of America; 6 Key Laboratory of Systems Bioengineering, Ministry of Education, Tianjin University, Tianjin, China; University of Freiburg, GERMANY

## Abstract

*Methylobacterium extorquens* AM1 is a facultative methylotroph capable of growth on both single-carbon and multi-carbon compounds. The ethylmalonyl-CoA (EMC) pathway is one of the central assimilatory pathways in *M*. *extorquens* during growth on C1 and C2 substrates. Previous studies had shown that ethylmalonyl-CoA mutase functioned as a control point during the transition from growth on succinate to growth on ethylamine. In this study we overexpressed *ecm*, *phaA*, *mcmAB* and found that upregulating *ecm* by expressing it from the strong constitutive *mxaF* promoter caused a 27% decrease in growth rate on methanol compared to the strain with an empty vector. Targeted metabolomics demonstrated that most of the central intermediates in the *ecm* over-expressing strain did not change significantly compared to the control strain; However, poly-β-hydroxybutyrate (PHB) was 4.5-fold lower and 3-hydroxybutyryl-CoA was 1.6-fold higher. Moreover, glyoxylate, a toxic and highly regulated essential intermediate, was determined to be 2.6-fold higher when *ecm* was overexpressed. These results demonstrated that overexpressing *ecm* can manipulate carbon flux through the EMC pathway and divert it from the carbon and energy storage product PHB, leading to an accumulation of glyoxylate. Furthermore, untargeted metabolomics discovered two unusual metabolites, alanine (Ala)–meso-diaminopimelic acid (mDAP) and Ala–mDAP–Ala, each over 45-fold higher in the *ecm* over-expressing strain. These two peptides were also found to be highly produced in a dose-dependent manner when glyoxylate was added to the control strain. Overall, this work has explained a direct association of *ecm* overexpression with glyoxylate accumulation up to a toxic level, which inhibits cell growth on methanol. This research provides useful insight for manipulating the EMC pathway for efficiently producing high-value chemicals in *M*. *extorquens*.

## Introduction

Methanol is emerging as an important renewable feedstock for the chemical industry with a global production of 53 million tons in 2011 and an expected annual production increase of 10–20% [1]. Biological conversion of methanol by methylotrophic bacteria provides a potentially important route for the production of industrial chemicals. *M*. *extorquens*, a facultative methylotrophic α-proteobacterium capable of using single carbon compounds and multiple carbon compounds as carbon and energy sources, has served as the best-characterized model organism for studying one-carbon metabolism [2,3]. *M*. *extorquens* is a promising biotechnological platform for utilizing C1 compounds to produce amino acids, single-cell protein, poly-β-hydroxybutyrate (PHB), and dicarboxylic acids [4–6]. However, success converting methanol into platform chemicals has been limited due to the regulatory complexity and metabolic redundancy of methylotrophic metabolism. In *M*. *extorquens* assimilation during methylotrophic metabolism involves three interlocked cycles: the serine cycle, the ethylmalonyl-CoA (EMC) pathway and the PHB cycle [7,8]. The main function of the EMC pathway is to regenerate glyoxylate for reincorporation into the serine cycle during the C1 assimilation [9–13].

The EMC pathway involves several CoA derivatives, such as crotonyl-CoA, ethylmalonyl-CoA, methylsuccinyl-CoA and mesaconyl-CoA, which are potential precursors for producing value-added chemicals [14–17]. For example, overexpression of heterologous thioesterases in *M*. *extorquens* led to the synthesis of the novel organic acid mesaconic acid [18]. In another study the engineered mevalonate pathway redirected carbonflux from acetoacetyl-CoA to generate the precursor of isoprene [19]. 1-Butanol was also produced from the EMC pathway from crotonyl-CoA by heterologous expression of two enzymes [20]. It is notable that these target chemicals derived from the EMC pathway have low production titers, ranging from 13.6 mg/L to 2.2g/L for methanol growth, relative to titers produced in *Escherichia coli* (from 8.6 g/L to 47 g/L) [18–22]. One straightforward approach to increasing the amount of products is to manipulate the expression of upstream genes to draw more flux through the EMC pathway. For instance, Hu et al. overexpressed the gene encoding crotonase, *croR*, to manipulate the production of a key intermediate crotonyl-CoA from 3-hydroxybutyryl-CoA, and divert its conversion into PHB. This approach increased production of 1-butanol from methanol by 50% [20]. However, unlike genes encoding the serine cycle, the genes coding for EMC pathway are not transcribed in large operons but are only loosely associated on the genome or not co-transcribed at all [23–25]. Recently, the *ecm* gene was shown to be a control point in the EMC pathway during the transition from C4 to C2 growth in *M*. *extorquens*, restricting consumption of ethylmalonyl-CoA to produce the downstream intermediates [26]. Moreover, *in vitro* enzyme activities for ethylmalonyl-CoA mutase, ß-ketothiolase, and methylmalonyl-CoA mutase, encoded by *ecm*, *phaA* and *mcmAB* respectively, had lower activities than the other EMC pathway enzymes during methanol growth [27]. Ecm had the lowest activity suggesting that *ecm* could also play a key role as a growth-limiting step for C1 growth [27,28]. Therefore, it is valuable to investigate whether overexpressing *ecm* could relieve the bottleneck of the EMC pathway, and accelerate the replenishment of glyoxylate during methanol growth. Regardless of the result, such a study would provide useful insight into improving the production of value-added chemicals in *M*. *extorquens*.

In this work, we overexpressed the *ecm*,*phaA*, and *mcmAB* with the strong promoter P*mxaF* to study the effect on the cell growth on methanol. When *ecm* was upregulated in *M*. *extorquens* the growth rate on methanol was significantly reduced compared to the control strain. We further investigated how the intracellular metabolome responded to *ecm* genetic perturbations and sought associations between metabolite changes and the growth defect.

## Materials and Methods

### Reagents

All chemicals including metabolite standards were purchased from Sigma-Aldrich (St. Louis, MO, USA) and Cambridge Isotope Laboratories, Inc (Andover, MA, USA). DNA polymerase, restriction enzymes, T4 ligase were purchased from Tankala (Dalian, China). Millpore water (Billerica, MA, USA) was used for the preparation of all media, buffers, standards, and sample solutions.

### Bacterial strains and growth conditions

The strains used in this study are listed in Table 1. *Escherichia coli* strains Top 10 and S17-1 were cultivated at 37°C in lysogeny broth medium [29]. *M*. *extorquens* was routinely cultured in the minimal medium described previously with 125 mM methanol in shake flasks [27]. 20 μM of final cobalt chloride was added to the culture medium [30]. Triparental matings between *E*. *coli* and *M*. *extorquens* were conducted on nutrient agar plates [31]. Antibiotics were supplied at concentrations as follows: tetracycline (Tet) at 20 μg/mL, and rifamycin (Rf) at 50 μg/mL. Growth curve assessments were carried out in biological triplicates. Tested *M*. *extorquens* strains were sub-cultured (0.5 mL) from tubes into 50 mL of minimal medium, then grown at 30°C on orbital shakers at 250 rpm. To assess the growth rates in the presence of glyoxylate, 0.5 mL of each culture was distributed into 50 mL fresh medium in 250 mL flasks containing different concentrations of glyoxylate. OD_600_ of the growing cultures was measured every 3 h until stationary phase. The growth rates presented represent the mean plus STDEV calculated from triplicate biological replicates, each inoculated from an independent pre-culture.

**Table 1:** *M*. *extorquens* strains, plasmids and primers used in this study.

**Strains**	**Genotype or relevant features**	**Source or reference**
Wide Type	Rifamycin-resistant strain	[2]
NG107	pCM80	[26]
NG115	pCM80 (PmaxF::*ecm*)	[26]
JY213	pCM80 (PmaxF::*phaA*)	this work
JY214	pCM80 (PmaxF::*mcmA*)	this work
JY215	pCM80 (PmaxF::*mcmB*)	this work
**Plasmids**	**Description**	
pCM80	*M*. *extorquens* expression vector, *mxaF* promoter; Tc^R^	[31]
pCM80-*ecm*	pCM80 containing *ecm* gene from *M*. *extorquens*	[26]
pCM80-*phaA*	pCM80 containing *phaA* gene from *M*. *extorquens*	this work
pCM80-*mcmA*	pCM80 containing *mcmA* gene from *M*. *extorquens*	this work
pCM80-*mcmB*	pCM80 containing *mcmB* gene from *M*. *extorquens*	this work
**Primers**	**Sequence (5’–3’)**	
*phaA*_pCM80_fw	ctcgagAAGCTT ATGGCAGCCAGTGAAGATATCGTC	this work
*phaA*_pCM80_rev	ctcgagGGATCC TCAGACCCGCTCGACACACATG	this work
*mcmA*_pCM80_fw	ctcgagAAGCTT ATGAGCTCCCGCATCCCCGATTTC	this work
*mcmA*_pCM80_rev	ctcgagGGATCC CTACTCGGCCGCCTGCCGTTC	this work
*mcmB*_pCM80_fw	ctcgagAAGCTT ATGGAAGATCGTCCGCTCGCCGA	this work
*mcmB*_pCM80_rev	ctcgagGGATCC GCGGCCTTGAGTGTTAGTCAGCC	this work

### DNA manipulations

The protein sequences of ß-ketothiolase, methylmalonyl-CoA mutase alpha subunit, and methylmalonyl-CoA mutase beta subunit were retrieved from GenBank with the following accession numbers: *phaA* [GenBank: ACS41411.1], *mcmA* [GenBank: ACS42853.1] and *mcmB* [GenBank: ACS40173.1]. The plasmid pCM80 containing the strong promoter of *mxaF* was used for overexpression [31,32]. Restriction sites *Hin*dIII and *Bam*HI were designed in front and behind of the sequences, respectively. After digestion, the fragments were ligated to *Hin*dIII/*Bam*HI-digested pCM80 for constructing plasmids pCM80-*phaA*, pCM80-*mcmA*, pCM80-*mcmB*.

### Extraction and measurement of the metabolome

Samples (10 mL) of mid-exponential cultures, at the OD_600_ of 0.8±0.1, were rapidly harvested by vacuum filtration using MILLEX-GP PES membrane filters (0.22 μm, 33 mm) and quickly washed with the growth medium (Millipore; Billerica, MA, USA) as described before [33]. Extraction of metabolites was carried out as previously published for *M*. *extorquens* with slight modification [34]. ^13^C3, ^15^N-alanine was added as the internal standard to correct for variation due to sample extraction and injection. 2 mL of boiling 4-(2-hydroxyethyl)-1-piperazineethanesulfonic acid (HEPES) buffered ethanol solution (75/25, v/v ethanol/water, pH 5.2) was added to a given sample and incubated at 100°C for 10 min. The extracted cell suspension was cooled on ice for 5 min, and then cell debris was removed by centrifugation at 5,000 rpm for 5 min. The cell-free metabolite extract was centrifuged at 14,000 rpm for 8 min. The supernatant was dried in a Rotational Vacuum Concentrator (Christ, Osterode, Germany) and stored at -80°C. For LC-MS analysis, each dried sample was dissolved in 100 μL of purified water. For GC-MS analysis, each sample was further derivatized in two steps. First, keto groups were methoximated by adding 50 μL of methoxyamine solution (25 mg/mL methoxyamine hydrochloride in pyridine) and incubated at 60°C for 30 min. Second, trimethylsilylation was performed by adding 50 μL of a TMS reagent (BSTFA/TMCS, 99:1) and incubated at 30°C for 90 min [35].

Sugar phosphates and acyl-CoAs were measured by LC-MS analysis. The sample analyzed on either an Agilent LC-QQQ-MS system (Agilent 1290 Infinity-6460, Agilent Technologies, Santa Clara, CA, USA), an Agilent LC-QTOF (Agilent 1290 Infinity-6530B, Agilent Technologies, Santa Clara, CA, USA) or a LC-QExactive-MS system (Thermo Fisher Scientific, Waltham, Massachusetts, USA). For LC-QQQ-MS, multiple reaction monitoring (MRM) precursor/product ion pairs were carried out as before [34]. For LC-QTOF, the m/z range was set to 50–1200 in centroid mode with a scan rate of 1.5 spectra/s. The ESI conditions were as follows: capillary voltage of 4000 V, fragmentor of 135 V, gas temperature of 300°C, nebulizer of 35 psig, gas flow of 10 L/min. For LC-QExactive-MS system, spray voltage was set to + 3.2 KV, sheath gas pressure was 35 arb, aux gas pressure was 10 arb, capillary temperature was 320°C and heater temperature was 350°C. Full MS (Resolution 70,000) and MS2 (Resolution 17,500) were carried out with the scan range from m/z 100 to 1000. The sample was separated on an Acquity UPLC BEH Amide column (150 × 2.1 mm, 1.7 μm; Waters, Milford, MA, USA). The mobile phase A was acetonitrile/water (2:98 v/v) with 0.1% ammonium hydroxide and 0.2% formic acid. The mobile phase B was acetonitrile/water (95:5 v/v) with 0.075% ammonium hydroxide and 0.1% formic acid. The linear gradient was as following: 0–1 min, 90% B; 1–2 min, 90%–65% B; 2–3 min, 65%–60% B; 3–6 min, 60%–50% B; 6–7.5 min, 50%–20% B; 7.5–9.5 min,20% B; 9.5–9.51 min, 20%-90% B; 9.51–15 min 90% B. The total run time was 15 min at 0.3 mL/min. The injection volume was 10 μL. All of the peaks were integrated by Qualitative Analysis B.06.00 software and Xcalibur (version 2.1).

Amino acids and carboxylic acids (except for glyoxylate) were determined by GC-MS. The derivatized samples were analyzed on Agilent 5975B/6890N GC-MS instrument (Agilent Technologies, Santa Clara, CA, USA). The column was a HP-5MS (30 m × 0.25 mm × 0.25 μm film; Restek, Bellefonte, PA, USA). Ultra high purity helium was used as the carrier gas in a constant flow mode of 1 mL/min, and 1 μL of a given sample was injected in split-less mode via an Agilent 7890 autosampler. The inlet and transfer line temperatures were set at 280°C. The temperature program started at 60°C with a hold time of 0.25 min, and then increased at 5°C /min to 280°C with a hold time of 10 min at 280°C. The ion source and quadrupole temperatures were set to 250°C and 150°C, respectively. Mass spectra were collected from m/z 40 to 500 at a rate of 3 spectra/s after a 4.5 min solvent delay. The chromatograms were processed using Agilent data analysis software.

For glyoxylate measurement, the sample preparation was as described above except that the amount of harvested cell culture was increased to 30 mL. The sample was separated on a ZIC-HILIC column (150×4.6 mm, 3 μm, 100 Å pore size; EMD Merck, Billerica, MA, USA) and detected by Agilent LC-QQQ-MS system (Agilent 1290 Infinity-6460). The mobile phase A was 10% (v/v) buffer in water and mobile phase B was 10% (v/v) buffer in acetonitrile. The buffer consisted of 200 mM formic acid adjusted to pH 4.0 with ammonium hydroxide solution [36]. The linear gradient was as follows: 0–1 min, 100%–90% B; 1–3 min, 90%–20% B; 3–10 min, 20% B; 10–10.01 min, 20%–100% B; 10.01–20 min, 100% B. The total run time was 20 min at 0.3 mL/min. The injection volume was 10 μL. All of the peaks were integrated by Agilent Qualitative Analysis B.06.00 software.

### Extracellular sample preparation and measurement

The samples for the determination of extracellular metabolites were removed from each flask when the culture had reached an OD_600_ of 0.8±0.1. 20 mL of the cell culture was filtered using a MILLEX-GP PES membrane filters (0.22 μm, 33 mm) and the supernatant was then lyophilized at approximately-45°C in a Free-dryer ALPHA 1–2 LD_plus_ (Christ, Osterode, Germany). 1 mL of purified water (ddH2O) containing 150 μL of 6N HCl was added to the dried culture medium and the resulting pH of the solution was less than 1 [13]. The acidified medium was applied to a C18 solid-phase extraction (SPE) column (1 mL; Phenomenex, CA, USA) to remove the salts. The C18 SPE column was preconditioned with 2 mL methanol and 2 mL water. After sample loading, 0.5 mL of water was added to wash the column. The metabolites were eluted with 2 × 0.5 mL of 50% methanol, and then 0.4 mL of 80% methanol. The eluate from the SPE column was collected into a 2-mL glass vial and dried in a vacuum centrifuge. Carboxylic acids and amino acids were measured as described above.

### Metabolomic data processing

For the targeted metabolome, the data were presented as the mean of three independent biological replicates. The significance of metabolite differences between the different strains was determined by t-tests (Origin 8.0) with a *p*-value less than 0.05 considered to be statistically significant. For the untargeted metabolome, four independent biological samples were collected at the OD_600_ of 0.8±0.1. LC-MS data was converted into mzML format using MS Convert software [37]. Data preprocessing and statistical analysis were performed with MZmine 2.10 and Metaboanalyst 3.0 [38,39]. For structure identification with MetFusion, the MS/MS peak list was used as input into MetFusion and then the molecule structures and scores were returned in SD files [40].

### PHB measurement

The PHB concentration in *M*. *extorquens* was determined by a gas chromatographic method developed by Braunegg et al. [41]. Briefly, the cell samples at the OD_600_ of 0.8±0.1 were centrifuged at 8000 rpm for 3 min and cells were suspended in a mixture of 2 mL methanol (3% H_2_SO_4_, v/v) and 2 mL chloroform, then heated at 100°C for 3.5h. 3mM benzoic acid was added as an internal standard. After cooling to room temperature, 1 mL H_2_O was added and the sample was shaken vigorously for 10 min and the organic phase was taken to analyze on GC-FID. The GC-FID instrument was a GC7900 capillary gas chromatograph with a flame ionization detector (Tian Mei, Shanghai, China), an Rtx^R^-1 column (30 m × 0.25 mm, 0.25 μm, Restek, Bellefonte, USA). The inlet and FID temperatures were set at 190°C and 220°C. The temperature program was as follows: 70°C with a hold time of 2 min, followed by an increase to 90°C at a rate of 5°C/min, then followed by an increase to 180°C at a rate of 15°C/min with no holding time in-between. Temperature was held at 180°C for 10 min.

### Formate detection in culture medium

Samples (1 mL) were taken from growing cultures (OD_600_ = 0.8±0.1) and pelleted by centrifugation at 8000 rpm for 2 min. Supernatants were used for formate assays as previous description [42]. 150μL volumes of supernatant were added to the reaction mixture containing 50 mM Tris HCl buffer, pH 8.0, 2 mM NAD, and 0.4 mg/mL yeast FDH (Sigma-Aldrich), in a total volume of 0.5 mL. The reaction mixtures were incubated at 37°C for 60 min, and the resulting NADH was measured spectrophotometrically at 340 nm.

### Ethylmalonyl-CoA mutase (Ecm) assay

50 mL of *M*. *extorquens* cells for enzyme activity were harvested and processed as reported previously [8]. Ecm activities was measured as reported by Good et al. previously [26].

## Results

### Comparison of the growth rate and yield of strains containing overexpressed EMC pathway genes

**Fig 1:**
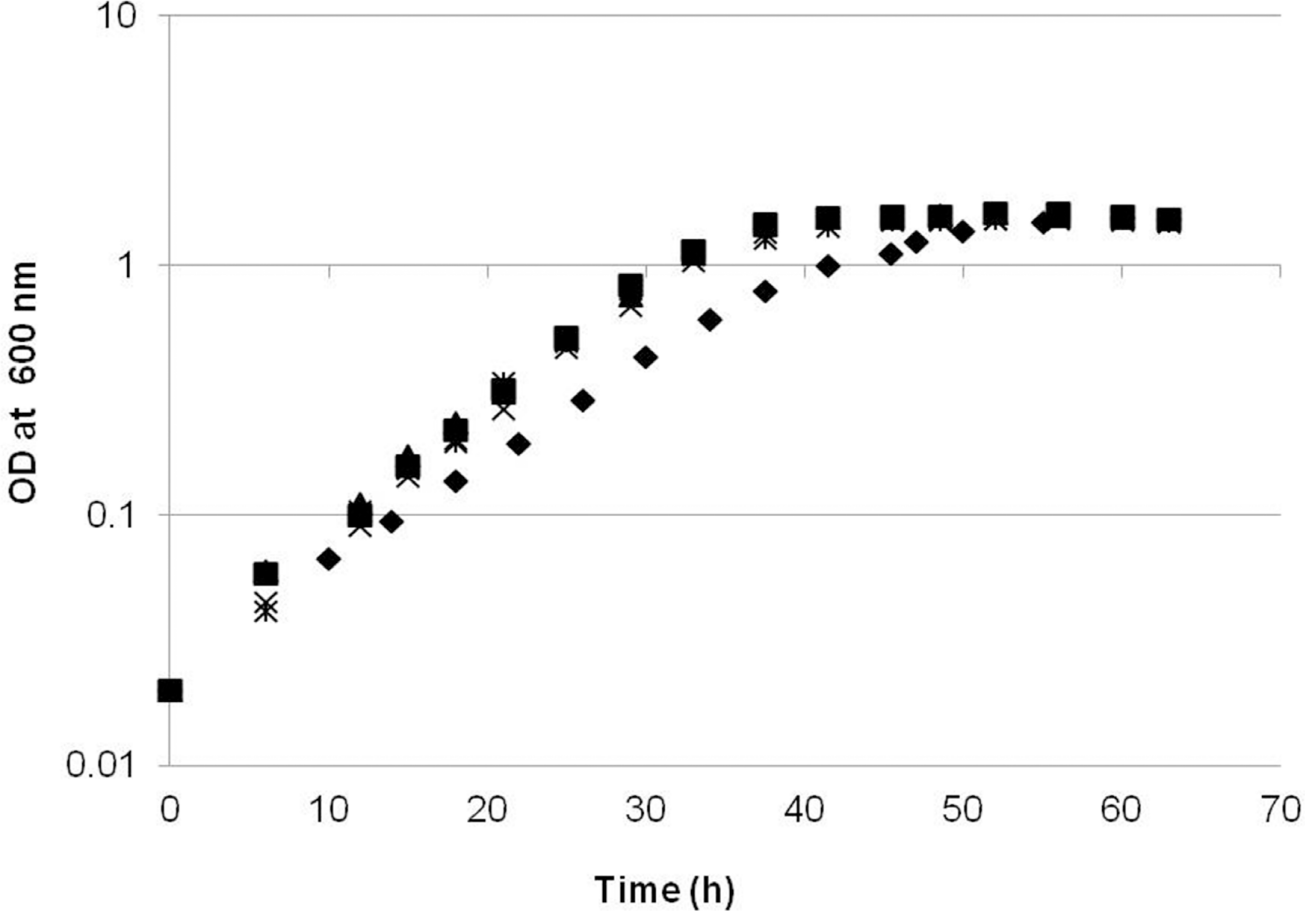
Growth on methanol of strains overexpressing EMC pathway genes. NG115 (WT/pCM80::*ecm*), diamonds; NG107 (WT/pCM80::), squares; JY213 (WT/pCM80::*phaA*), triangles; JY214 (WT/pCM80::*mcmA*), crosses; JY215(WT/pCM80::*mcmB*), stars. The growth rates presented for each strain were the mean calculated from three independent biological replicates.

Since previous studies had suggested that a part of the EMC pathway might be a bottleneck for growth on C1 compounds, we overexpressed four key genes (*ecm*, *phaA* and *mcmAB*) using the strong promoter of *mxaF* (Table 1) and tested the effect on cell growth in batch culture with 125mM methanol as carbon source. As shown in Fig 1, compared to the growth curve of empty vector background (the NG107 strain), the strains overexpressing *phaA*, *mcmA* and *mcmB* had no significant growth change. In contrast, the NG115 strain overexpressing *ecm* had a specific growth rate of 0.085 h^-1^, which is 27% lower than that of the NG107 strain (0.117 h^-1^). Moreover, the growth yield was not significantly different among the strains of JY213, JY214, JY215, NG115 and NG107 (Fig 1). The specific activity for ethylmalonyl-CoA mutase (Ecm) in cell-free extracts was 4-fold higher in the NG115 (44±4 nmol^.^mg^-1.^min^-1^) strain compared to the control strain (11±1 nmol^.^mg^-1.^min^-1^).

### Targeted metabolomics of the NG107 and NG115 strains

**Fig 2:**
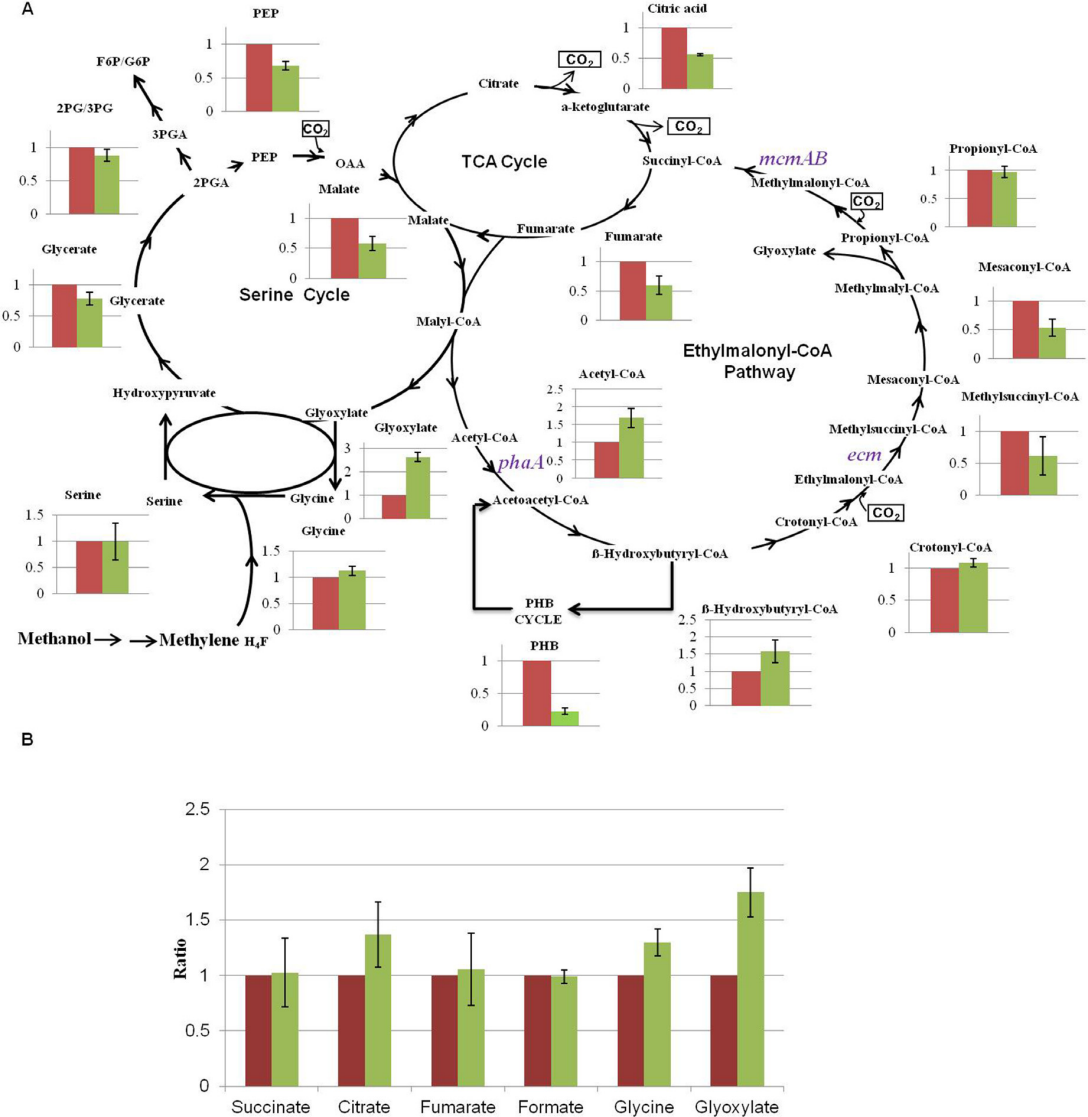
Comparison of the intermediates involved in the serine cycle, the TCA cycle and the ethylmalonyl-CoA pathway between the NG107 strain (Red) and NG115 strain (Green) grown on methanol. (A) Intracellular metabolites. (B) Supernatant metabolites. The y axis of (A) and (B) is the ratio of the abundance of the NG115 strain to the NG107 strain. The average value for the NG107 strain was set to 1. Data show the mean with error bars indicating standard deviation calculated from three independent biological replicates.

To determine why the NG115 strain grew slower than the NG107 strain, we first investigated the central metabolic intermediates in *M*. *extorquens* by using LC-MS and GC-MS. *M*. *extorquens* uses the serine cycle, the EMC pathway, and the PHB cycle for one-carbon assimilation [9–13]. For the EMC pathway, the amounts of crotonyl-CoA and propionyl-CoA in the NG115 strain were similar to that in the NG107 strain (Fig 2A). Mesaconyl-CoA was decreased by 1.8-fold in the NG115 strain and acetyl-CoA and 3-hydroxybutyryl-CoA were increased 1.7-fold and 1.6-fold in the NG115 strain compared to the NG107 strain. For the serine cycle, most metabolites such as glycerate, 2-/3-phosphoglycerate, serine, and glycine did not show obvious differences between the NG107 and NG115 strain. However, glyoxylate, an integral intermediate connecting the EMC pathway and the serine cycle, was found to be 2.6-fold higher in the NG115 strain compared to the NG107 strain. Moreover, PHB levels decreased 4.5-fold in the NG115 strain. In addition, the abundances of fumarate and citrate were 1.7 and 1.8-fold lower in the NG115 strain, respectively. We also measured several acids in the culture supernatant of the NG115 and NG107 strain as shown in Fig 2B. Only glyoxylate was found to increase by 1.8-fold in the supernatant of NG115 strain, suggesting it was excreted during the growth.

### Untargeted metabolomics of the NG107 and NG115 strains

**Fig 3:**
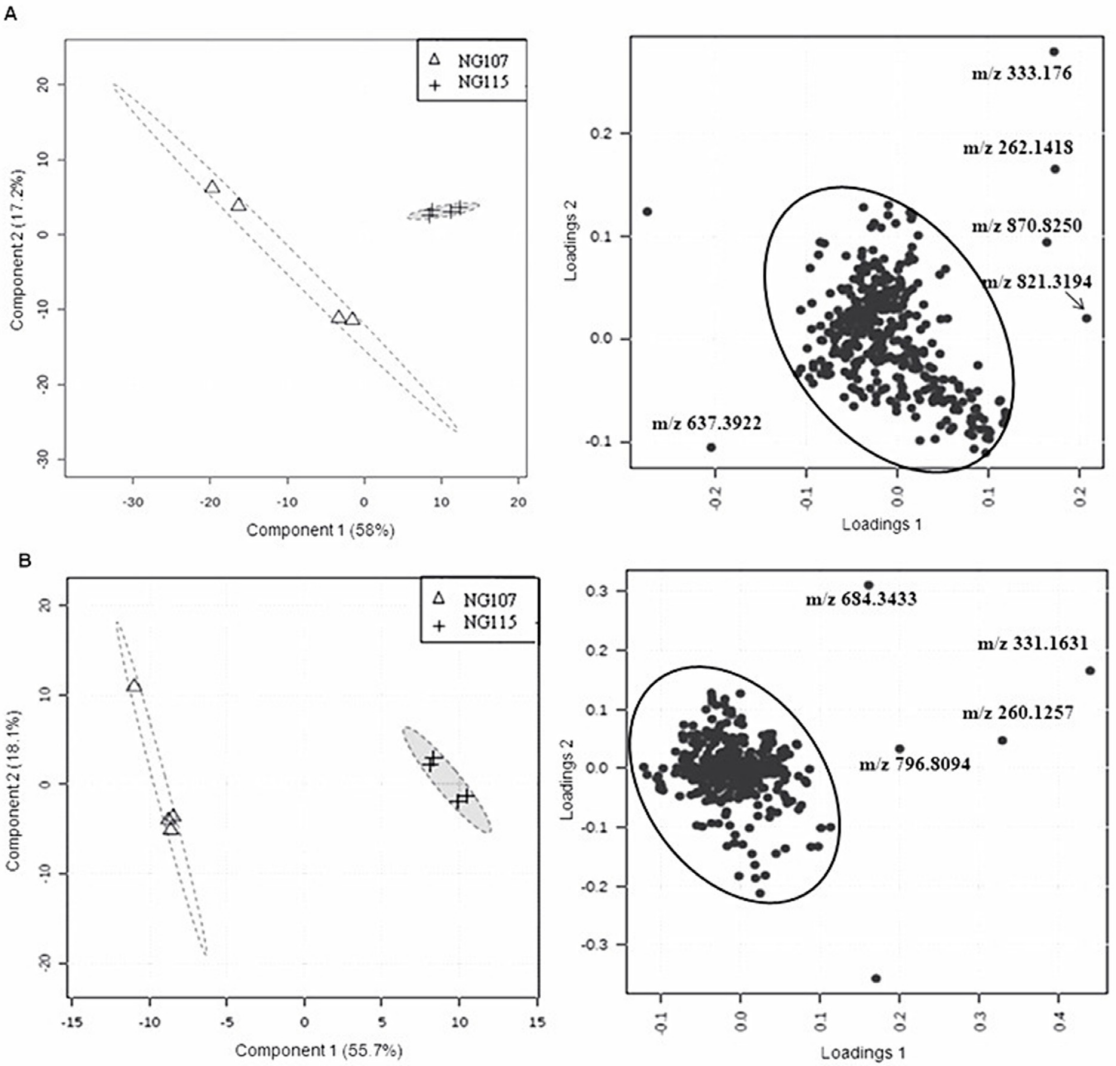
Untargeted metabolomics of the NG107 and NG115 strains grown on methanol. The metabolomic data was processed by PLS-DA. (A) The score and loading plot of the metabolome analyzed by LC-MS in the positive mode. (B) The score and loading plot of the metabolome analyzed by LC-MS in the negative mode. One scattered dot was not labeled with m/z in the Fig 3A and 3B because one replicate in the NG107 strain was not as reproducible as other three replicates. Data was acquired from four independent biological replicates.

To assess the differences of other metabolites between the strain NG115 and NG107, partial least squares discrimination analysis (PLS-DA) method was used, which is a supervised clustering approach and reduces the dimensionality of the raw data while preserves most of the variances in a 2- or 3-dimensional map. Examination of the scores plot in Fig 3A left and 3B left showed that the strain NG115 was clearly separated from the NG107, which accounted for total 75.2% and 73.8% of the variability in the data. The loading plot in Fig 3A right and 3B right revealed that the main variables responsible for this separation between the NG115 and the NG107 strain were the scattered dots outside the circle. Nine features were further confirmed (Fig 3 and Table 2). Two of these features were distinct in the positive scan mode of LC-MS, m/z 262.1395 and 333.1769 (negative mode was m/z 260.1257 and 331.1630) were over 45-fold higher in the NG115 strain compared to the NG107 strain (Table 2). The detailed information on these two features including predicted elemental composition and the ratio of the abundance are shown in Tables 2 and 3. We also detected the change of these two features in other strains overexpressing *phaA*, *mcmA*, *mcmB*. Compared to the NG107 strain, there were no significant differences of m/z 260.1257 and 331.1630 in JY215, JY214 and JY213 strains (Table 3).

**Table 2:** The list of distinct metabolites present in the NG115 strain measured in the positive and the negative mode.

**LC-MS [M+H]**			
**Compounds**	**m/z**	**Predicted elemental composition**	**Ratio of the abundance of NG115 to NG107**
Putative Ala-mDAP	262.1395	C_10_H_19_N_3_O_5_	53.3±4.6
Putative Ala-mDAP-Ala	333.1769	C_13_H_25_N_4_O_6_	47.6±8.4
Unknown	870.8250	C_36_H_13_C_l3_O_10_S_5_	13.9±2.1
Unknown	821.3194	C_49_H_40_N_8_O_5_	8.9 ±0.4
Unknown	637.3922	C_39_H_56_O_5_S	0.103±0.004
**LC-MS [M-H]**			
Putative Ala-mDAP	260.1257	C_10_H_19_N_3_O_5_	30.7±5.3
Putative Ala-mDAP-Ala	331.1630	C_13_H_25_N_4_O_6_	22.8±4.7
Unknown	684.3433	C_35_H_59_NO_6_S_3_	15.8±3.6
Unknown	796.8094	C_28_H_3_ClN_4_O_15_S_4_	15.3±4.1

**Table 3:** The ratio of the abundance of m/z 262.1395 and m/z 333.1769 between the over-expressed strains (JY213, JY214, JY215 and NG115) and the NG107 strain.

m/z [M+H]	Ratio			
	JY215/NG107	JY214/NG107	JY213/NG107	NG115/NG107
262.1395	0.98±0.14	0.78±0.14	2.42±0.31	53.30±4.60
333.1769	1.13±0.19	0.84±0.23	1.79±0.37	47.60±8.40

### A dose-dependent correlation between glyoxylate addition, growth rate and mass features accumulation

**Fig 4:**
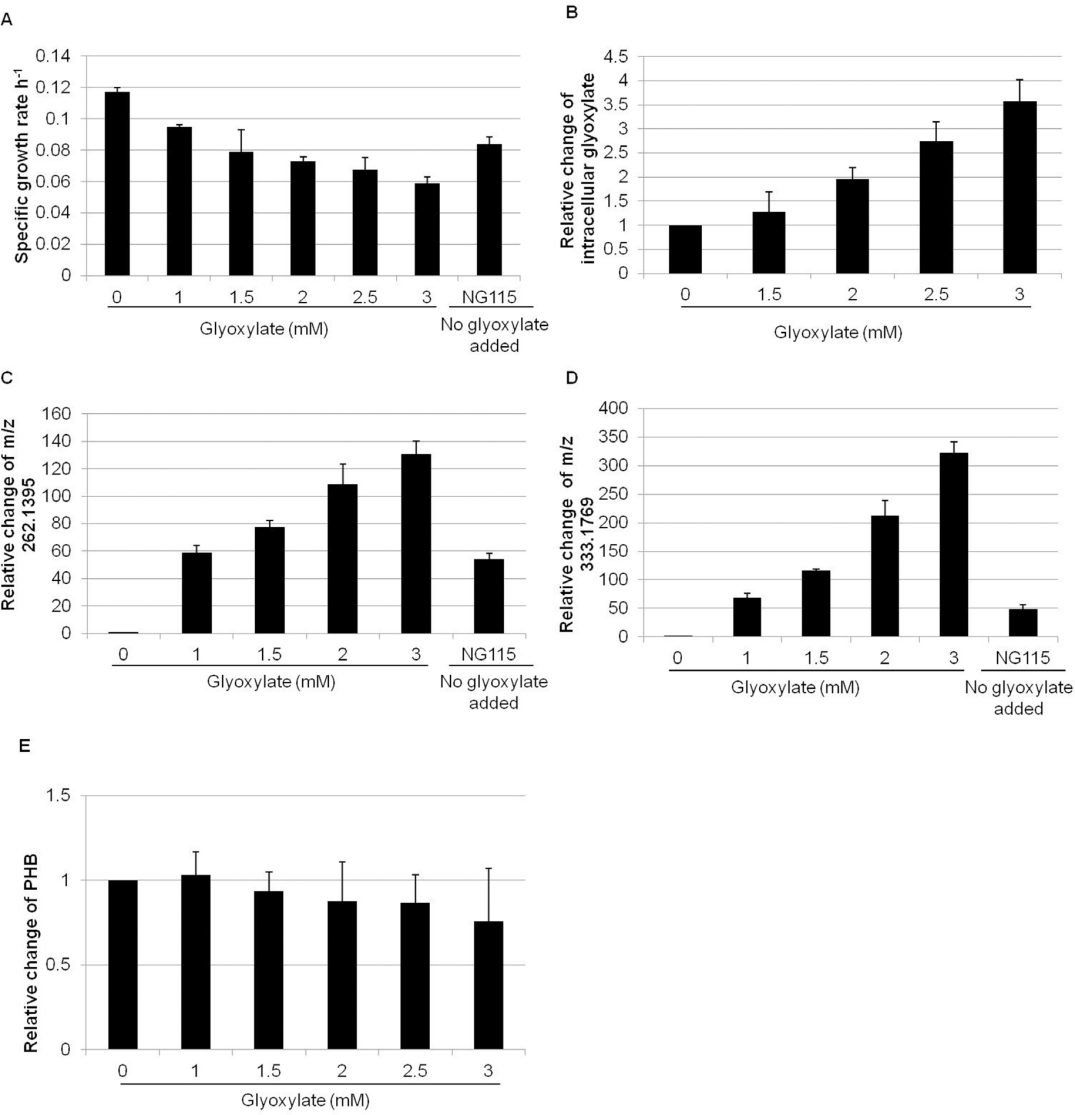
The effect of the addition of glyoxylate on the NG107 strain compared to the growth of the NG115 strain with no added glyoxylate. (A) The growth rate of the NG107 strain was reduced with increasing concentration of glyoxylate; (B) The abundance of intracellular glyoxylate increased upon addition of glyoxylate to the growth medium. The y axis is the ratio of intracellular glyoxylate between strain NG107 with addition of glyoxylate to the growth medium and strain NG107 without addition of glyoxylate. X axis is the concentration of glyoxylate in the growth medium; (C) and (D) Increase in the amount of m/z 262.1395 and 333.1769 with addition of glyoxylate; (E) PHB concentration does not change with addition of glyoxylate. The y axis is the ratio of PHB between strain NG107 with addition of glyoxylate to the growth medium and strain NG107 without addition of glyoxylate. The x-axis is the concentration of glyoxylate in the growth medium; Data is the mean with standard deviation calculated from three independent biological replicates.

In order to evaluate whether the accumulation of glyoxylate could be responsible for the growth defect of the NG115 strain and also contribute to the accumulation of the discovered metabolites, we added different concentrations of glyoxylate to the medium of the NG107 strain. As shown in Fig 4A, the growth rates dropped from 0.117 h^-1^ to 0.059 h^-1^ in a glyoxylate dose-dependent manner and the abundance of intracellular glyoxylate increased significantly upon the addition of 3 mM glyoxylate to the growth medium (Fig 4B). Two mass features corresponding to m/z 262.1395 and 333.1769 significantly increased up to 130-fold and 321-fold respectively compared to the control with no addition of glyoxylate (Fig 4C and 4D). In contrast, PHB did not change significantly with the addition of glyoxylate (Fig 4E).

### Identification of unknown metabolites with m/z 262.1395 and 333.1769

**Fig 5:**
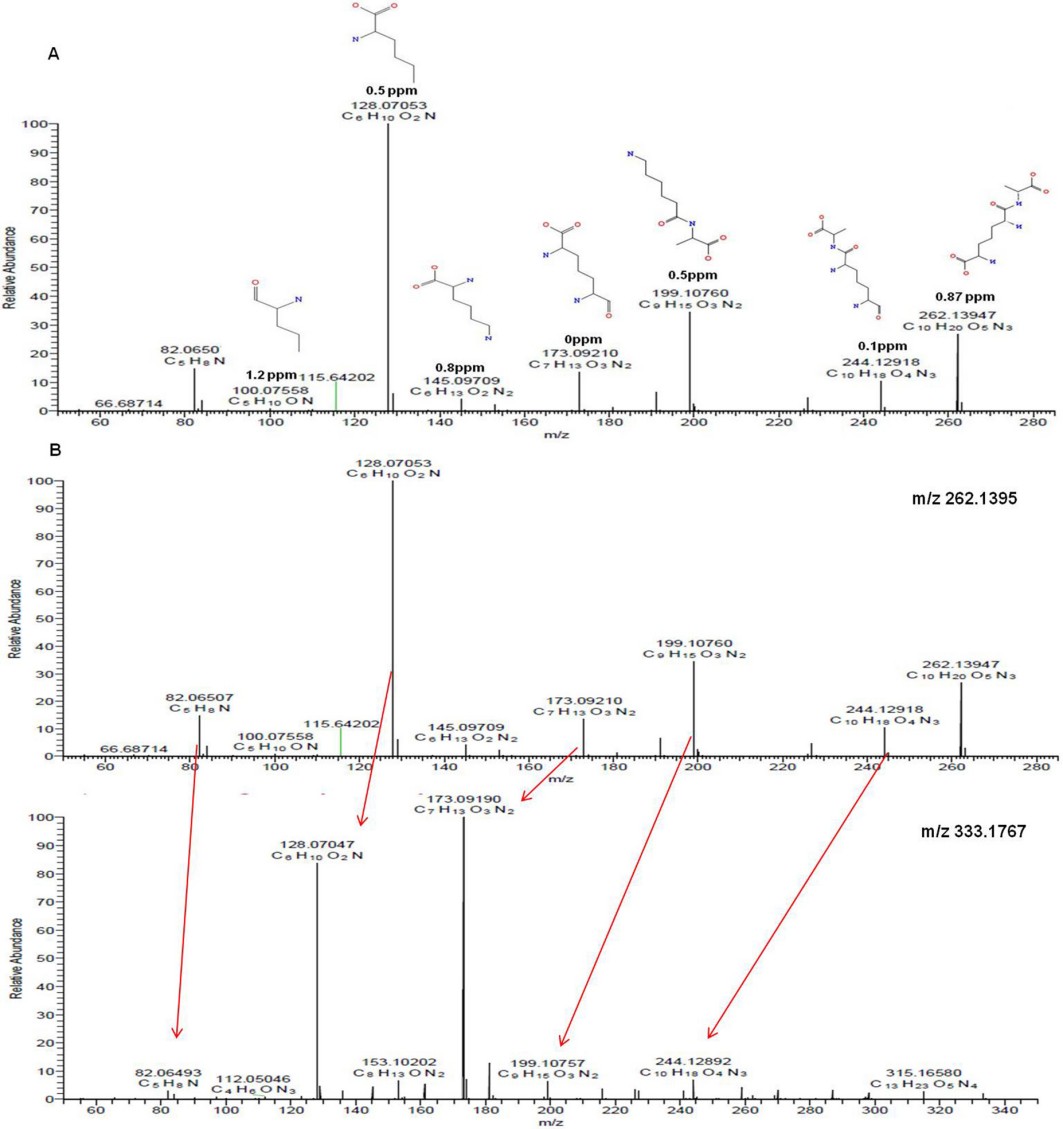
The fragmental structures of m/z 262.1395 and m/z 333.1767 were predicted by MetFusion and manual analysis. (A) The fragmental structures of m/z 262.1395 predicted by MetFusion analysis. The mass accuracy between the theoretical mass and experimental mass is shown below the structure. (B) The comparison of MS/MS between m/z 262.1395 and m/z 333.1767.

**Fig 6:**
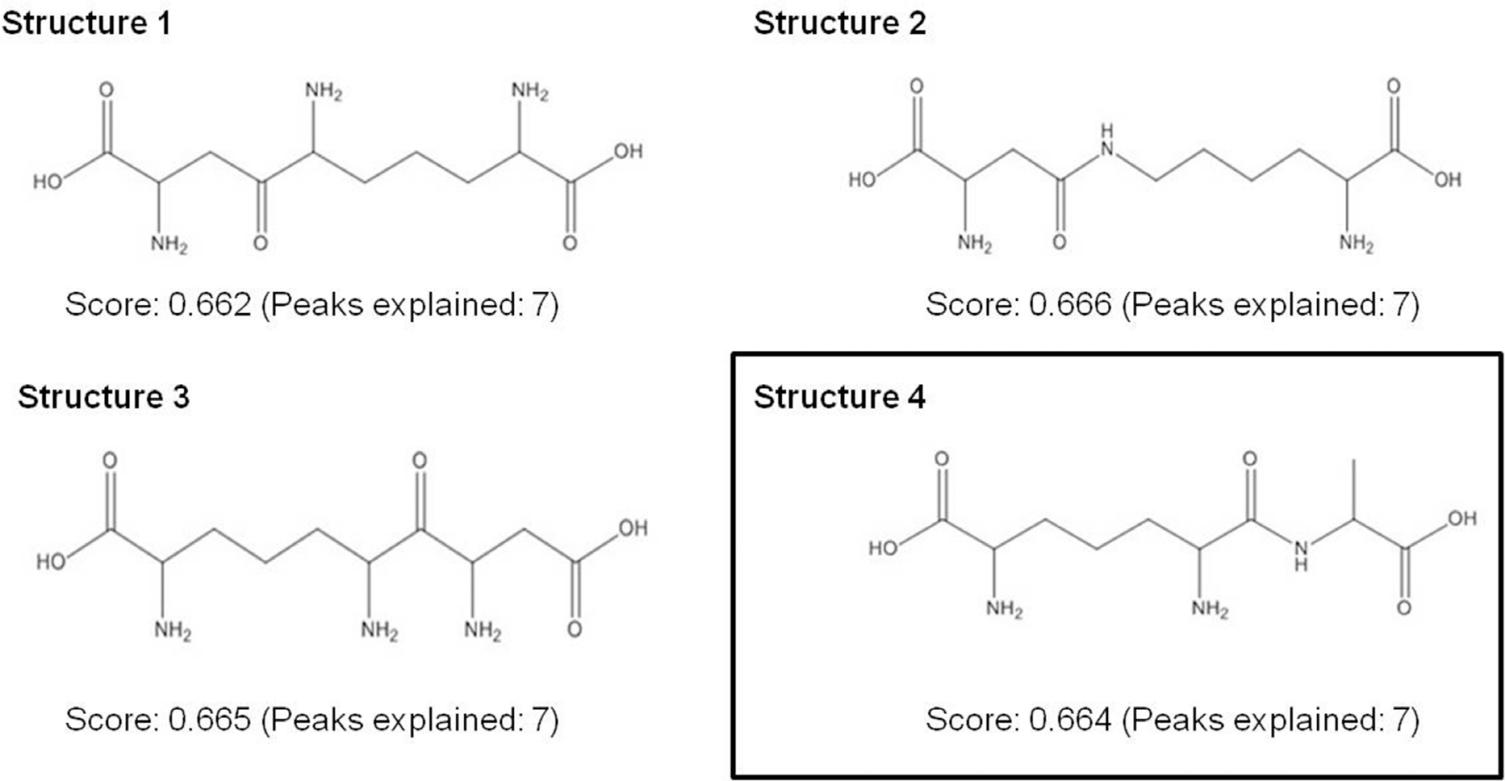
The structure of m/z 262.1395 (C_10_H_19_N_3_O_5_) was identified as Ala-mDAP according to the prediction of MetFusion analysis against PubChem and manual search against MetaCyc database.

To determine the putative structures of the m/z 262.1359 and 333.1769, MS/MS was carried out on a high resolution MS. As shown in Fig 5A, MetFusion analysis was first used to predict the structure and fragmental structures of m/z 262.1395. For example, the product ion at m/z 128.0753 was suggested to be C_6_H_10_O_2_N and the product ion at m/z 199.1076 corresponded to C_9_H_15_O_3_N_2_, which was interpreted as loss of CO_2_ and NH_2_ from the precursor ion of m/z 262.1395. The product ion at m/z 173.0921 was proposed to be the loss of 89 mass units, consistent with loss of C_3_H_7_NO_2_ from the precursor ion. Then the results obtained from MetFusion were searched against Pubchem and Chemspider databases to find the most probable candidates (Fig 6). We further took those candidates through a manual search in the MetaCyc and KEGG databases to look for possible biological roles. Finally, m/z 262.1395 was identified as Ala-mDAP as shown in the Fig 6 (Structure 4). For the m/z 333.1767, its MS/MS was very close to MS/MS of Ala-mDAP, i.e. m/z 262.1395 was a loss of C_3_H_5_NO from m/z 333.1767, which was the alanine residue (Fig 5B). Therefore, we propose m/z 333.1767 as a tripeptide of Ala-mDAP-Ala.

## Discussion

*M*. *extorquens* grows on one-carbon compounds using the serine cycle for carbon assimilation. This metabolism requires the EMC pathway to replenish the serine cycle with glyoxylate. Recently, Good et al. demonstrated that ethylmalonyl-CoA mutase encoded by *ecm* was a key control point in the EMC pathway during the carbon switch from C4 to C2 [26]. Smejkalová et al. also found that ethylmalonyl-CoA mutase had much lower activity compared to other enzymes involved in the EMC pathway, suggesting the *ecm* gene could be a potential metabolic control point in methanol growth as well [27].

In this work, we used overexpression as a tool to investigate the function of *ecm* and other EMC pathway genes for the EMC pathway during methanol growth. *M*. *extorquens* overexpressing *ecm* grew slower than the control strain. Recent reports have demonstrated that a tradeoff between the growth rate and an overexpressed gene can be due to (1) the burden of expression of the protein, and/or (2) accumulation of a toxic metabolite [43–45]. However, no obvious growth defect was observed when the NG115 strain was grown on succinate (data not shown) and when the strains overexpressing *phaA* and *mcmAB* were grown on methanol, suggesting that the growth defect was not due to the burden of protein expression. We further hypothesized that toxic intermediates could have accumulated as a result of overexpressing of *ecm*. The central metabolic intermediates such as serine, glycine, crotonyl-CoA, methylsuccinyl-CoA and propionyl-CoA did not change significantly, suggesting that the methylotrophic network was quite robust and flexible [24,46]. A similar result was observed in *sga*, *mclA1*, and *gcvP* mutant strains of *M*. *extorquens*, in which many serine cycle and EMC pathway intermediates were not significantly changed [13]. This concept of metabolite pool robustness has been observed in other microbes such as *Saccharomyces cerevisiae*, in which key intermediates and cofactors were generally robust under various perturbations and single enzyme deletions elicited only local accumulation of the metabolites [47,48].

The primary exception to metabolite robustness observed in our studies was PHB, which significantly decreased in the NG115 strain while its precursor 3-hydroxybutyryl-CoA increased. PHB is a carbon and energy storage compound, and the extent to which it accumulates in the cell varies depending on growth substrate [49]. Previous results have suggested that when a shift occurs from succinate to methanol, *M*. *extorquens* can redirect flux from PHB synthesis to the EMC pathway with a concomitant decrease in expression of genes for PHB synthesis [8]. Results from a switchover from succinate to ethylamine growth at the early phase of transition also suggested a similar result [26]. In this study we tested the hypothesis that overexpressing *ecm* could repartition the carbon flux from PHB synthesis and draw more flux through the EMC pathway, which could result in the accumulation of a toxic intermediate such as glyoxylate. Overexpressing *ecm* resulted in PHB level was decreased 4.5-fold and branch point metabolite of 3-hydroxybutyryl-CoA was increased 1.6-fold. It also resulted in a build-up of glyoxylate during methanol growth. Notably, this observation was in contrast to the reported finding that glyoxylate did not accumulate when *ecm* was overexpressed in a transition of C4 to C2 growth. In the previous research, the growth rate of the NG115 strain increased from 0.024 h^-1^ to 0.071 h^-1^ 8 h after the switchover [26]. It was proposed that the change in growth rate occurred because during C2 metabolism glyoxylate condenses with acetyl-CoA, generating malate, which is then converted to oxaloacetate [26]. During the transition of C4 to C2 when *ecm* was overexpressed to relieve the bottleneck of EMC pathway, excess glyoxylate-consuming capacity existed and glyoxylate did not accumulate. However, in the current study, the NG115 strain grew slower than the NG107 strain on methanol. One possible explanation is higher flux went through the EMC pathway would produce surplus glyoxylate, which in turn might overwhelm the capacity of the serine cycle to consume it and was excreted to the medium [12,24,50]. It was reported that glyoxylate was toxic at mM levels and can inhibit the cell growth [51]. Therefore it is likely that the accumulation of glyoxylate caused growth inhibition on methanol when *ecm* was overexpressed. One possible strategy for remediation of toxic glyoxylate accumulation is to use a synthetic glyoxylate assimilation module to convert glyoxylate into a non-toxic cellular metabolite [52].

Untargeted metabolomics identified two compounds with m/z 262.1395 and 333.1769 that accumulated markedly in the NG115 strain, but not in other overexpressed strains (i.e. JY213, JY214 and JY215). These two compounds were identified as Ala-mDAP and Ala-mDAP-Ala, based on their accurate mass, their fragmentation, and MetFusion prediction. It has been reported that Ala-mDAP could be produced via hydrolyzing peptidoglycan by D-glutamyl-meso-diaminopimelate amidase encoded by *mpaA* in *Escherichia coli* [53,54]. It has been also reported that the intermediates of peptidoglycan recycling could be increased due to cytotoxicity [55] and another dipeptide (Glu-mDAP) was produced during antibiotic stress in *Bacillus subtilis* [56]. We compared the protein sequence of *mpaA* and related genes against the genome of *M*. *extorquens*, and found that the putative peptidoglycan-binding protein encoded by META1_5226 had 36.6% sequence identity to the 43 amino acids C-terminal sequence of the peptidase M14 encoded by a *mpaA* homolog. More importantly, the NG107 strain treated with glyoxylate resulted in increasing the intracellular glyoxylate, then in turn caused strong accumulation of Ala-mDAP and Ala-mDAP-Ala, suggesting that glyxoylate could be a potential inducer for synthesizing these two peptides. Therefore, these results suggest an unknown link between generation of these di-and tri-peptides and glyoxylate accumulation. Additional knowledge is required to clarify the function of these two peptides.

Insight into manipulating the EMC pathway could be vital to engineering *M*. *extorquens* as a platform for producing value-added chemicals. The current results indicate that overexpressing *ecm* can potentially accelerate the generation of toxic glyoxylate, which further results in the accumulation of unexpected metabolites and defective growth. These results suggest that the coordinative manipulation of multiple genes in EMC pathway and serine cycle will be necessary to reduce the risk of toxic metabolite buildup and to balance cell growth and chemicals synthesis.
